# A small neighborhood well-organized: seasonal and daily activity patterns of the community of large and mid-sized mammals around waterholes in the Gobi Desert, Mongolia

**DOI:** 10.1186/s12983-021-00412-1

**Published:** 2021-05-17

**Authors:** Battogtokh Nasanbat, Francisco Ceacero, Samiya Ravchig

**Affiliations:** 1 Faculty of Tropical AgriSciences, Czech University of Life Sciences Prague, Prague, Czechia; 2 Institute of Biology, Mongolian Academy of Sciences, Ulaanbaatar, Mongolia; 3 School of Arts and Sciences, National University of Mongolia, Ulaanbaatar, Mongolia

**Keywords:** Activity overlap, Arid environments, Camera-trapping, Ecological segregation, Resource competition, Waterholes

## Abstract

**Background:**

Animal communities have complex patterns of ecological segregation at different levels according to food resources, habitats, behavior, and activity patterns. Understanding these patterns among the community is essential for the conservation of the whole ecosystem. However, these networks are difficult to study nowadays, due to anthropic disturbances and local extinctions, making it difficult to conclude if segregation patterns are natural or human-induced. We studied ecological segregation in a community of large and mid-sized mammals in the Great Gobi Desert, a remote arid area free from recent extinctions and human disturbances. Activity patterns of 10 sympatric mammal species were monitored around 6 waterholes through camera-trapping over a two-year period, and analyzed them primarily through circular statistics.

**Results:**

Complex patterns of spatial, seasonal, and daily segregation were found. Overlap in seasonal activity was detected in only 3 of the 45 possible pairs of species. Four species used the waterholes all-year-round, while others peaked their activity during different periods. The Bactrian camel showed continuous daily activity, the grey wolf had bimodal activity, and the argali and Siberian ibex were diurnal, while the others had nocturnal peaks during different hours. Daily and spatial overlap were both detected in only 6 of the 45 pairs. Only one species pair (snow leopard and Eurasian lynx) showed an overlap at two levels: seasonal and daily. Climate and moon phase significantly affected the activity of certain species.

**Conclusions:**

Altogether, the results showed complex patterns of ecological segregation at different levels in the use of the key resource in arid environments: waterholes. These results are important for understanding the biology of these species under natural conditions, as well as potential changes in altered ecosystems, and may help to design conservation strategies.

## Background

Animals must adjust their daily and seasonal rhythms, and allocate their activity budget according to their physiological, metabolic, and social needs. Furthermore, these rhythms must be adapted to the environment, presence and availability of key resources, and inter- and intra-specific interactions and conflicts (numerous examples can be found in [20, 40, 46, 48]). The species show a variable degree of flexibility in their activity patterns, strongly influenced by the surrounding conditions, in such a way that patterns can differ significantly between populations of a given species under different environmental constraints [30]. In other words, one cannot understand the behavior and ecology of a population without understanding how it is affected by the surrounding environment (from daylength and season, to weather conditions [10];), key resources, and the zoological community in the area.

Activity levels are well-studied for many mammal species, due to its importance as behavioral and ecological metrics. However, interactions in the activity patterns within an ecological community are poorly understood, since it is difficult to quantify activity in the field in a consistent, cost-effective, and non-invasive way [66]. Moreover, biological aspects within each species in the community (like species richness, different animal densities and detectability in complex and heterogeneous habitats, differential use of resources and niche occupancy, etc. [31];) may compromise the collection of accurate and comparable data for each of the individual species within the community.

Camera-trapping is a widespread and useful tool for determining the activity patterns of large and mid-sized mammals [51, 64]. Camera-trapping can be implemented within a wide variety of ground and climatic conditions, and can be used to collect information about elusive species in difficult terrains where other field techniques are likely to fail [65]. Among such situations, placing camera traps at water sources in arid environments has been shown to be one of the most effective survey designs for various studies, including those focused on activity patterns [23, 33].

Mountainous areas of the Mongolian Gobi Desert are an excellent location to study interactions between the activity patterns of large and mid-sized mammals for several reasons. Firstly, it supports strong populations of some of the most threatened mammal species in Central Asia [5, 14, 17, 61, 68], but the number of species is moderate, which facilitates the interpretation of such results. Secondly, the community includes similar numbers of prey species (khulan, argali, Siberian ibex, goitered gazelle, and tolai hare) and predators (grey wolf, snow leopard, red fox, and Eurasian lynx), which can be expected to interact regarding their activity patterns. Moreover, the activity patterns of predators and prey may also be affected by the moon phase, as previously shown by Griffin et al. [28] and Harmsen et al. [32]. Precipitation is so low in the area that water is the dominant controlling factor for biological processes [50], and water points have become essential resources for most of the occurring species. Indeed, the distance to water points is known to be an important habitat requirement for several charismatic species in the region, like Bactrian camels [71], khulan [62], snow leopard [43] and brown bear (locally known as Gobi bear: [69]). Water points in the Great Gobi “A” Strictly Protected Area (GGASPA) are a matrix of ephemeral oases that routinely fluctuate, which influences the movement patterns of fauna in the region [72]. Moreover, precipitation in the area is highly unpredictable and variable throughout the year, which allows for studying the influence of weather on activity patterns. Finally, the orography allows for predicting the accesses for wildlife, which decreases the probability of missing events, as may happen in more heterogeneous habitats.

Currently, little is known about the daily and seasonal rhythms of water point usage by large and mid-sized mammals in the Gobi Desert, as well as inter-species interactions at water points without human influence (including the absence of livestock). This study aims to determine how large and mid-sized mammals use the scarce water sources in the area, detect seasonal and daily overlaps in the use of this resource among species, and understand the influence of environmental factors (climate and moon phase) on the activity patterns observed for each species in the community inhabiting such an extreme and fragile ecosystem. Considering that the species in the area can develop their natural strategies freely, due to the lack of human influence, low levels of activity overlap were predicted. This low overlap is expected to be especially apparent between predator and prey species.

## Methods

### Study area

**Fig. 1:**
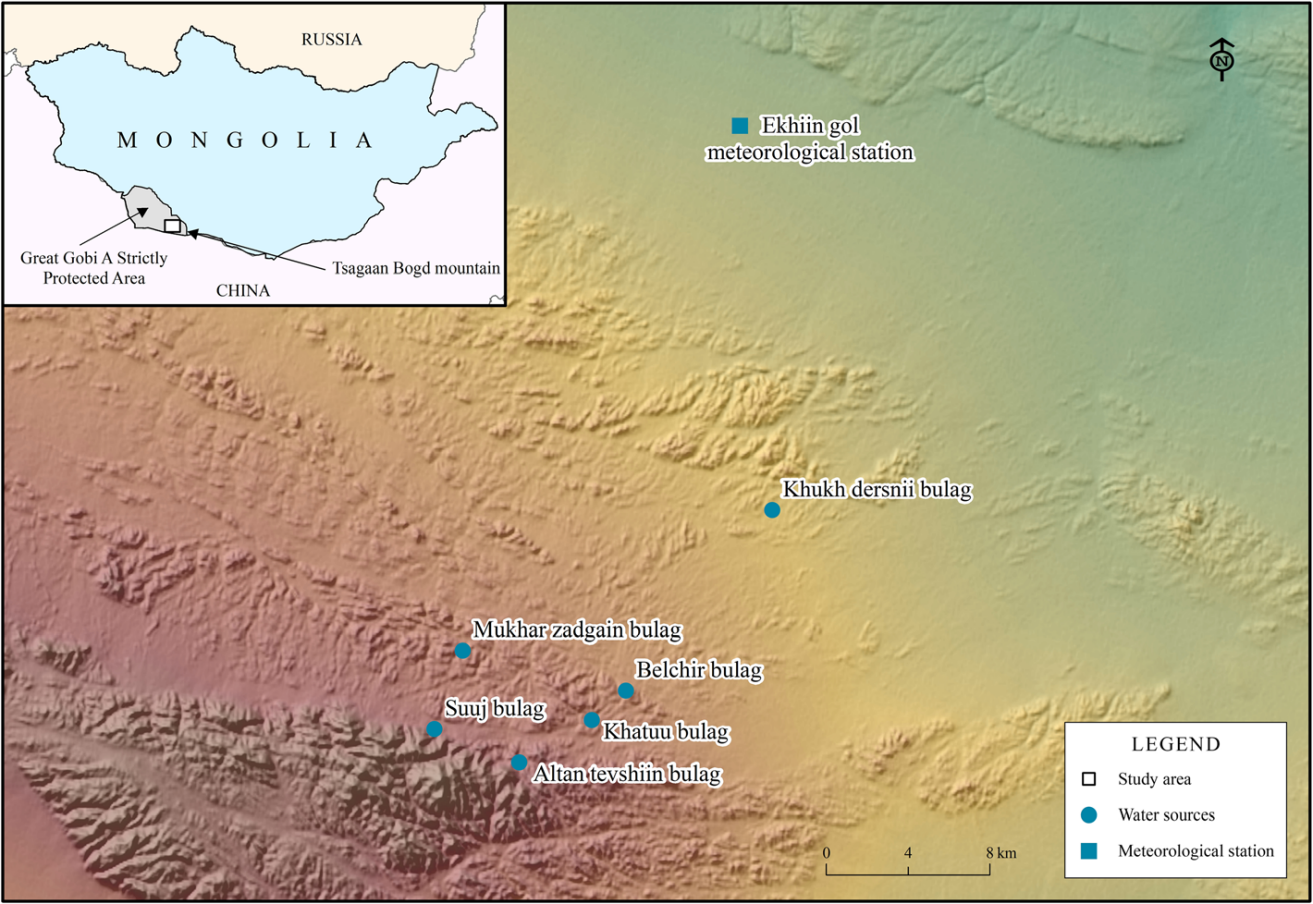
Studied water sources in the eastern part of the Great Gobi A Strictly Protected Area

The study area belongs to the Gobi biogeographical region, and the locations studied lie inside the GGASPA, in the south-western part of Mongolia and adjacent to its international boundary with China (Fig. 1). The area is protected since 1975, to protect representative samples of the Central Asian desert and semi-desert ecosystems together with their unique flora and fauna, and has been included in the World Network of Biosphere Reserves since 1990, being one of the largest biosphere reserves in the world [59]. A special focus was placed on large mammals, particularly wild Bactrian camel (*Camelus ferus*), brown bear (*Ursus arctos*; see [38, 73] for recent proposed changes in its taxonomical status), snow leopard (*Panthera uncia*), argali wild sheep (*Ovis ammon*), and Asiatic wild ass or khulan (*Equus hemionus*), all of which are listed in the Mongolian Red List of Mammals and the Mongolian Red Book ([17, 68]; Table 1). The GGASPA covers 4.419 million ha, with elevations ranging from 525 to 2683 m.a.s.l., and encompasses both large, mostly unvegetated depressions, extensive hilly areas, and several mountain ranges (Atas Bogd, Tsaagan Bogd, Eej Uul, and Edren). It is one of the most extreme arid areas in Central Asia [9], being part of the so-called “cold deserts of Central Asia” with a highly continental climate [75]. Since the Trans-Altai Gobi does not receive much rain, neither in summer nor in winter, the scarce water sources are thus essential for wildlife. Detailed climatic data from the eastern part of the protected area is only available from the Ekhiin Gol meteorological station, at the northern boundary of the study area (Fig. 1). Maximum and minimum aerial temperatures recorded in the area ranged from − 34 to + 40 °C, and ground temperatures from − 33 to + 70 °C. During winter, there is usually little to no snow in the mountains. Most of the precipitation falls from July to August, usually in the form of showers, in small amounts. The average precipitation is around 60 mm, with annual variations from 30 to 140 mm [29, 35]. During the study period, May 2015 to May 2017, the monthly average air temperature ranged from − 14.6 to + 25.5 °C, monthly average relative humidity ranged from 27.0 to 78.0%, average wind speed ranged from 1.80 to 4.17 m/s, and total precipitation over the 2 years was 88.5 mm.

**Table 1:** Species detected during the study, ranked by the number of independent events, percentage of the total independent events recorded, and the mean (±SE) daily trapping rate. The IUCN [34] and national [17] conservation statuses are also indicated

Species	IUCN Conservation Status (Global / Mongolia)	Independent events (n / %)	Mean daily trapping rate (× 100)
*Equus hemionus* – Khulan	NT / EN	1416 / 29.76	28.69 ± 1.40
*Ursus arctos* – Brown bear	LC / CR	1105 / 23.22	22.43 ± 1.62
*Vulpes vulpes* – Red fox	LC / NT	771 / 16.20	17.39 ± 0.76
*Lepus tolai* – Tolai hare	LC / LC	402 / 8.45	9.08 ± 0.55
*Ovis ammon* – Argali	NT / EN	306 / 6.43	6.46 ± 0.48
*Capra sibirica* – Siberian ibex	LC / NT	299 / 6.28	6.05 ± 0.47
*Canis lupus* – Grey wolf	LC / NT	227 / 4.77	4.86 ± 0.60
*Camelus ferus* – Bactrian camel	CR / EN	116 / 2.44	2.53 ± 0.35
*Lynx lynx* – Eurasian lynx	LC / NT	57 / 1.20	1.29 ± 0.20
*Panthera uncia* – Snow leopard	VU / EN	57 / 1.20	1.27 ± 0.20
*Otocolobus manul* – Pallas’s cat	NT / NT	1 / 0.02	0.02 ± 0.02
*Gazella subgutturosa* – Goitered gazelle	VU / VU	1 / 0.02	0.02 ± 0.02

### Data collection

The study was conducted at six different natural springs, without human influence, in the Tsagaan Bogd Mountain, located in the Eastern part of the protected area. All locations provided water consistently throughout the study. Passive infrared triggered Scoutguard HCO SG565F camera traps monitored the use of these water sources by large and mid-sized mammals. The study site was the experimental unit, and all the pictures collected from the cameras at each site were analyzed together. Five to eight cameras were deployed at each of the water sources, depending on the orography of each location (i.e., the number of routes available for the large mammals living in the area to access each water source). Thus, the average distance between each camera ranged between 60 to 120 m. The cameras were functional from May 13th, 2015 until May 15th, 2017. A total of 38 camera traps were mounted on natural rocks, bushes, and sticks. Cameras were generally orientated towards the north or south, to reduce solar glare, and set at a height of 50–70 cm from the ground, as recommended in previous studies [31]. Data collection was conducted every second month over the study period, before the batteries were depleted.

### Statistical analyses

Consecutive photographs of the same species at the same study site were considered as independent events when there was at least a one-hour interval between them [11]. This way, the 25,404 pictures collected with the presence of any of the studied species were reduced to 4758 independent events. The number of independent events for each species, and the percentage of the total independent events recorded, were summarized with the software CameraSweet [67], while the mean (±SE) daily trapping rate was calculated with the ZSL – Camera Trap Data Management and Analysis Package v.3.0.0 [3].

Circular statistics were used to unravel the seasonal and daily overlap in the activity of the studied species. These analyses were carried out in the software Oriana v4.02 (Kovach Computing Services, Anglesey, Wales). First, the mean vector (μ) and the circular standard deviation (CSD) was calculated for each species and context (seasonal and daily). Thereafter, the Rayleigh’s Uniformity Test [25] was used to calculate if the data for each species (μ ± CSD) was uniformly distributed (i.e., when the test is significant, the data is clumped around a certain date or time). This test is based on the length and direction of the mean vector, and may not be significant when the species has a bimodal daily activity. Thus, the Rao’s Spacing Test [42] was also calculated for the daily activity, which is based on the uniformity of the spacing between adjacent points. In summary, if any of these tests is significant, one can conclude that the seasonal or daily activity of the species is not uniform. Finally, the Watson’s U^2^ Test [42] was used to determine if two species have similar seasonal or daily activity patterns. Significant results meant that the two species have different activity patterns.

Spearman’s correlation coefficients were used to identify spatial overlap among the studied species in the area. The frequency of occurrence of each species at each study site, normalized by effort, was used for this purpose, after calculation with CameraSweet software [67].

With regards to the lunar activity patterns, the hourly activity pattern for 11 days, centered on the Full moon and the New moon, was calculated for each species. Thereafter, the square root of the sum of the squared differences in frequency between both lunar phases was calculated using CameraSweet. The greater the difference, the more active a species is during one phase of the moon compared to the other phase [67].

Finally, correlations were used to show the relationships between the monthly average weather conditions during the study and the number of events per month (corrected by the sampling effort). None of the variables tested were normally distributed (Shapiro-Wilk’s test), and thus a non-parametric test was chosen (Spearman’s ranked correlation). These tests were conducted in IBM© SPSS© version 25.

Only the species with an adequate number of observations (see [39]) were used in the previously described analyses.

## Results

The khulan was the most frequently observed species around the scarce waterholes of GGASPA over the two-years study period, with 1416 independent events and a mean daily trapping rate of 28.69 (Table 1). The brown bear, red fox, tolai hare, argali, Siberian ibex, grey wolf, Bactrian camel, Eurasian lynx, and snow leopard followed in their observed frequencies, while Pallas’s cat and goitered gazelle were observed only once. Thus, these latter two species were excluded from further analyses.

**Fig. 2:**
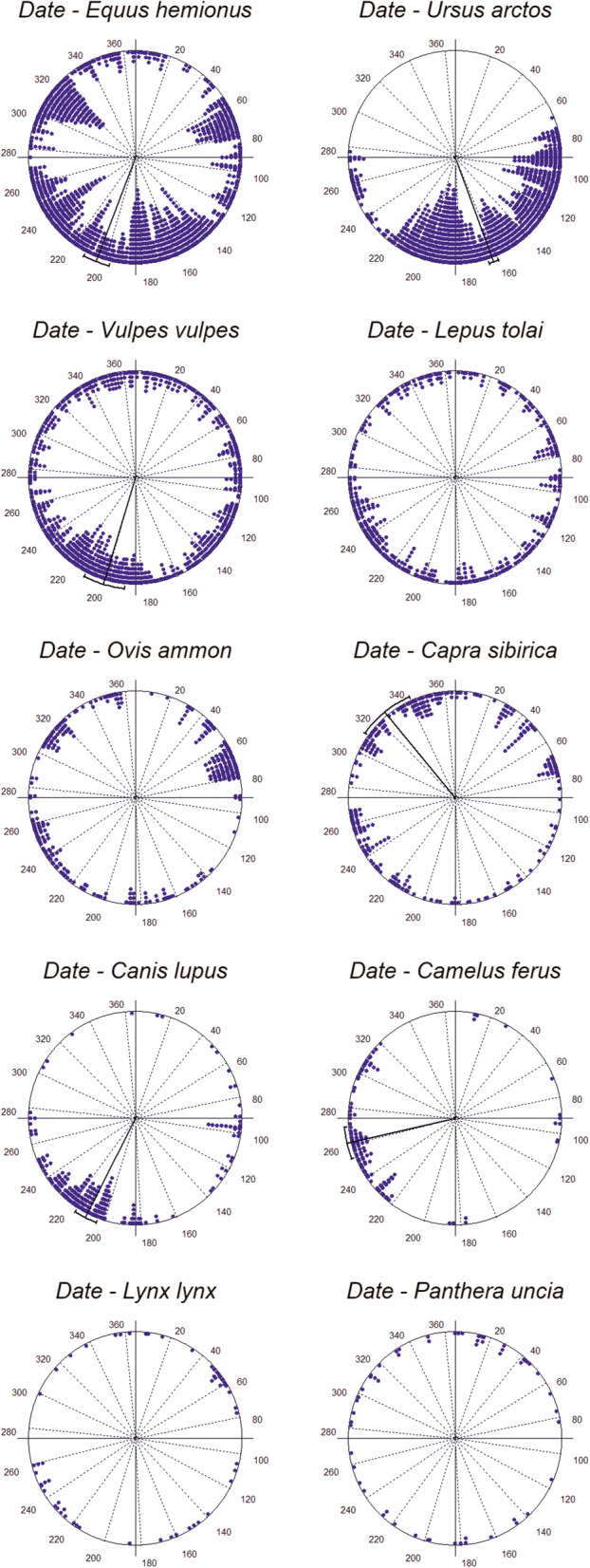
Circular histogram showing the seasonal activity of the community of large and mid-sized mammals around waterholes in the Great Gobi A Strictly Protected Area. Each dot in the graphs correspond to each observed independent event, clustered in bars for each of the 365 days of the year. The closer to the center, the higher activity of a species at a given date. Mean activity and circular 95% confidence interval are displayed when these are significant, and absent when the observations are uniformly distributed all-year-round

All-year-round, four species showed a uniform distribution of seasonal observations (Rayleigh Test was not significant, Fig. 2), and thus continuous use of the water resources throughout the year by the tolai hare, argali, Eurasian lynx, and snow leopard. The other six species studied showed a clumped distribution. The brown bear showed a peak of activity (μ ± CDS) around the first half of June (161.9 ± 22.4; Z = 607.503; *p* < 0.001); red fox around mid-July (199.4 ± 47.8; Z = 51.211; *p* < 0.001); khulan in the second half of July (203.5 ± 45.4; Z = 122.553; *p* < 0.001); grey wolf around the end of July (209.7 ± 23.7; Z = 115.800; p < 0.001); Bactrian camel around mid-September (260.7 ± 23.6; Z = 59.870; p < 0.001); and Siberian ibex around mid-November (325.1 ± 45.0; Z = 27.101; p < 0.001). Watson’s U Test identified few overlaps in the seasonal use of the waterholes between the argali-Eurasian lynx, Siberian ibex-snow leopard, and Eurasian lynx-snow leopard; thus, all these species share a uniform distribution of seasonal activity patterns. All the other possible species pairs showed significantly different seasonal activity patterns (Table 2).

**Table 2:** Seasonal overlap in the use of waterholes by the 10 studied mammals. For an adequate interpretation of these results, it is recommended to see this table together with Fig. 2. Values shown correspond to the Watson’s U Test, which identifies differences between the distribution of the observations for two species. Non-significant relationships (in bold) indicate overlap between two species, while significance implies seasonal segregation. ***, **, and * indicate significance at the 0.001, 0.01, and 0.05 level, respectively

	Brown bear	Red fox	Tolai hare	Argali	Siberian ibex	Grey wolf	Bactrian camel	Eurasian lynx	Snow leopard
**Khulan**	9.286***	1.029***	0.607***	2.285***	3.231***	4.878***	3.307***	0.483***	0.600***
**Brown bear**		9.588***	7.362***	11.957***	12.670***	6.470***	6.653***	2.223***	2.217***
**Red fox**			0.422***	2.144***	2.827***	2.674***	3.600***	0.396***	0.525***
**Tolai hare**				0.965***	1.641***	3.690***	2.247***	0.198*	0.409***
**Argali**					0.786***	5.465***	2.472***	**0.161**	0.295**
**Siberian ibex**						5.418***	2.238***	0.518***	**0.121**
**Grey wolf**							3.319***	1.439***	1.849***
**Bactrian camel**								1.072***	1.340***
**Eurasian lynx**									0.252*

**Fig. 3:**
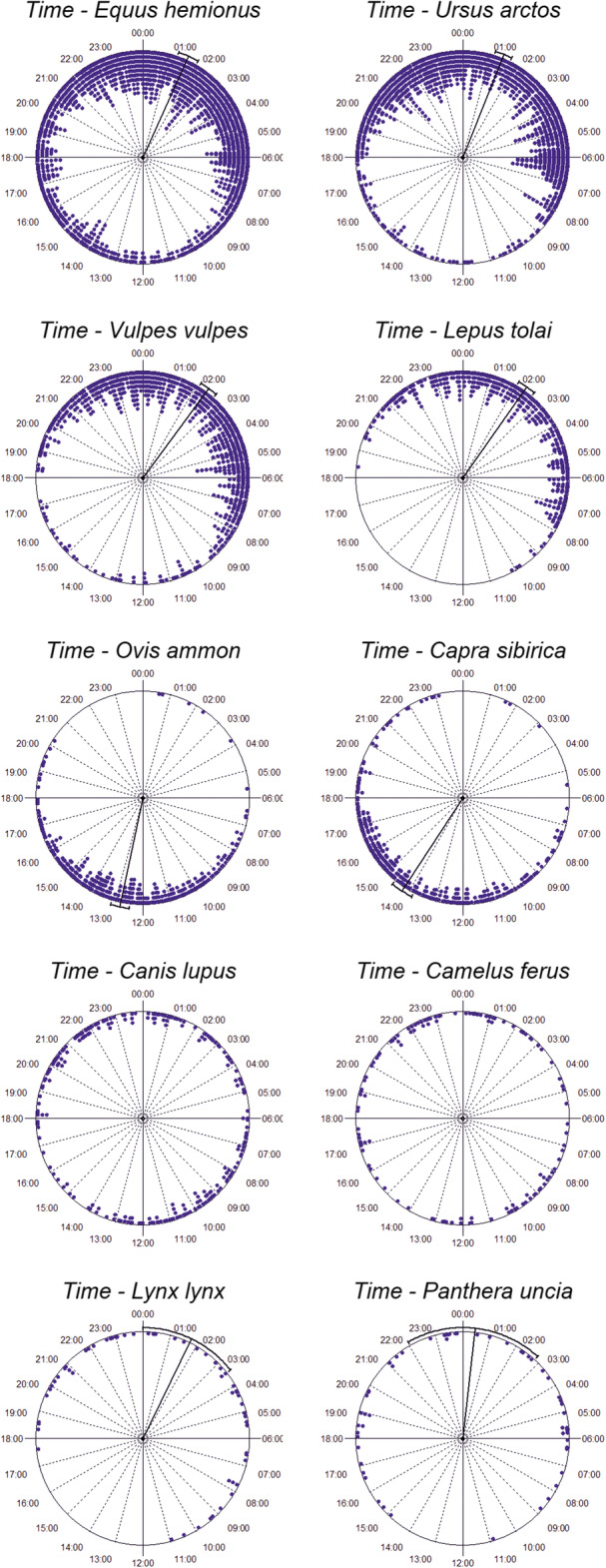
Circular histogram showing the daily activity of the community of large and mid-sized mammals around waterholes in the Great Gobi A Strictly Protected Area. Each dot in the graphs correspond to each observed independent event, clustered bars for each 10-min interval. The closer to the center, the higher the activity of a species at a given time interval. Mean activity and circular 95% confidence interval are displayed when these are significant, and absent when the observations are uniformly distributed over the entire day

Regarding the daily patterns, only the Bactrian camel showed a uniform pattern, being observed throughout day with the same probability (Fig. 3). All the other species showed a clumped distribution (Table 3). The argali (12:47 ± 1:28; Z = 169.117; *p* < 0.001) and Siberian ibex (14:13 ± 1:45; Z = 129.078; p < 0.001) were the only species with diurnal activity. The snow leopard (00:25 ± 3:00; Z = 4.973; *p* = 0.009), brown bear (01:26 ± 2:17; Z = 263.720; p < 0.001), khulan (01:37 ± 2:40; Z = 199.351; p < 0.001), Eurasian lynx (01:43 ± 2:37; Z = 8.714; p < 0.001), tolai hare (02:20 ± 1:40; Z = 185.984; p < 0.001), and red fox (02:25 ± 2:06; Z = 228.487; p < 0.001) had nocturnal activity, all of them showing peaks within a couple of hours during the middle of the night. The grey wolf was the only species showing a bimodal activity pattern (Rao’s Spacing Test: U = 145.823; *p* < 0.01), with greater activity during dawn and dusk, moderate activity at night, and lower activity during the day. In general, most pairs of species showed a lack of overlap in their daily activity. However, an overlap was detected between the grey wolf-Bactrian camel (the two species with a relatively uniform distribution), khulan-Eurasian lynx, khulan-snow leopard, brown bear-Eurasian lynx, Bactrian camel-snow leopard, and Eurasian lynx-snow leopard.

**Table 3:** Daily overlap in the use of waterholes by the 10 studied mammals. For an adequate interpretation of these results, it is recommended to see this table together with the Fig. 3. Values shown correspond to the Watson’s U Test, which identifies differences between the distribution of the observations for two species. Non-significant relationships (in bold) indicate overlap between two species, while significance implies seasonal segregation. ***, **, and * indicate significance at the 0.001, 0.01, and 0.05 level, respectively

	Brown bear	Red fox	Tolai hare	Argali	Siberian ibex	Grey wolf	Bactrian camel	Eurasian lynx	Snow leopard
**Khulan**	0.531***	0.747***	1.444***	13.533***	11.321***	1.315***	0.724***	**0.082**	**0.142**
**Brown bear**		0.509***	1.252***	16.161***	14.240***	2.008***	1.096***	**0.083**	0.201*
**Red fox**			0.443***	14.385***	13.461***	2.547***	1.577***	0.195*	0.339**
**Tolai hare**				13.200***	12.689***	3.424***	2.244***	0.494***	0.612***
**Argali**					0.824***	4.231***	3.076***	3.083***	2.865***
**Siberian ibex**						4.112***	2.629***	2.853***	2.187***
**Grey wolf**							**0.068**	0.289**	0.228*
**Wild camel**								0.264*	**0.181**
**Eurasian lynx**									**0.069**

The studied species also showed a high degree of spatial segregation (Table 4). The frequency of occurrence normalized by effort at each studied location was only correlated for the following pairs: khulan-tolai hare, khulan-Bactrian camel, khulan-grey wolf, brown bear-Siberian ibex, grey wolf-tolai hare and Bactrian camel-tolai hare. In other words, the khulan, tolai hare, grey wolf and Bactrian camel showed a certain ranked preference for the same waterholes, while the other species showed independent patterns of site preferences.

**Table 4:** Spatial overlap in the use of waterholes by the 10 studied mammals. Values shown correspond to Spearman’s Rho correlation. Significant relationships (in bold) indicate overlap between two species. ***, **, and * indicate significance at the 0.001, 0.01, and 0.05 level, respectively

	Brown bear	Red fox	Tolai hare	Argali	Siberian ibex	Grey wolf	Bactrian camel	Eurasian lynx	Snow leopard
**Khulan**	−0.371	0.429	1.000***	0.600	−0.486	0.883*	0.853*	0.257	−0.600
**Brown bear**		0.429	−0.371	−0.771	0.886*	− 0.618	− 0.383	− 0.429	0.429
**Red fox**			0.429	−0.143	0.257	0.118	0.441	−0.371	− 0.314
**Tolai hare**				0.600	−0.486	0.883*	0.853*	0.257	−0.600
**Argali**					−0.771	0.756	0.353	0.543	−0.543
**Siberian ibex**						−0.530	− 0.588	−0.086	0.771
**Grey wolf**							0.621	0.647	−0.353
**Wild camel**								−0.177	− 0.765
**Eurasian lynx**									0.371

Only the snow leopard and Eurasian lynx showed an activity pattern moderately influenced by the lunar phase (Table 5). Both predators showed greater activity around the studied waterholes during the full moon. The differential activity during Full vs. New moon was much lower in the other studied species, but most of them still showed a tendency for being more active during the full moon.

**Table 5:** Differences in the activity patterns of large and mid-sized mammals in the Gobi Desert, around the Full and New moon. The preferred lunar phase for each species is indicated. The difference index is based on the square root of the sum of the squared differences in frequency between both lunar phases

Species	Preferred lunar phase	Difference index
Snow leopard	Full moon	0.33
Eurasian lynx	Full moon	0.27
Bactrian camel	Full moon	0.19
Grey wolf	Full moon	0.16
Siberian ibex	Full moon	0.13
Argali	Full moon	0.12
Red fox	New moon	0.10
Tolai hare	Full moon	0.10
Brown bear	New moon	0.10
Khulan	New moon	0.05

Finally, weather conditions did not affect the seasonal activity patterns observed in the red fox, argali, Siberian ibex, and Bactrian camel. However, the khulan, tolai hare, grey wolf and Eurasian lynx were significantly more active when the air temperature and precipitation were higher (Table 6). Only the brown bear, the only species in the area that hibernates, showed a strong positive influence of temperature and precipitation on the monthly average activity, being inactive in those months with an average temperature below zero. Relative humidity correlated only with the activity of the brown bear, grey wolf, Eurasian lynx, and snow leopard, usually negatively (greater activity with lower humidity), but relative humidity was positively correlated with the activity of the snow leopard. Wind had no effect on the activity patterns observed.

**Table 6:** Spearman’s correlation coefficients (ρ) between the average monthly weather conditions over the study period and the mean occurrence of independent events (corrected by the sampling effort). ***, **, and * indicate significance at the 0.001, 0.01, and 0.05 level, respectively

	Air Temperature (°C)	Relative Humidity (%)	Wind Speed (m/s)	Precipitation (mm)
**Khulan**	0.706 *	−0.287^ns^	0.287^ns^	0.641 *
**Brown bear**	0.894 ***	−0.838 **	0.458^ns^	0.584 *
**Red fox**	0.510 ^ns^	−0.538^ns^	0.049^ns^	0.189^ns^
**Tolai hare**	0.690 *	−0.574^ns^	0.368^ns^	0.490^ns^
**Argali**	0.021 ^ns^	0.182^ns^	−0.203^ns^	0.377^ns^
**Siberian ibex**	−0.427 ^ns^	0.567^ns^	−0.518^ns^	−0.053^ns^
**Grey wolf**	0.692 *	−0.664 *	0.280^ns^	0.431^ns^
**Bactrian camel**	0.236 ^ns^	−0.145^ns^	0.384^ns^	0.355^ns^
**Eurasian lynx**	0.615 *	−0.853 **	0.406^ns^	0.139^ns^
**Snow leopard**	−0.381 ^ns^	0.628 *	−0.307^ns^	−0.086^ns^

## Discussion

This two-year camera-trap survey highlights a high degree of behavioral segregation within the community of large and mid-sized mammals regarding their use of waterholes in the Gobi Desert, a key resource in these arid environments. Segregation was observed at different levels: spatially, daily, and seasonally, and even for certain differential behavioral patterns related to the moon phase, altogether allowing the co-existence of a relatively large number of species for such a harsh environment. This confirms the initial prediction of low activity overlaps in an environment free of humans and livestock, which is rather rare nowadays. The second prediction is also confirmed, since the only overlap between a predator and a potential prey species was detected for the snow leopard, with the khulan and Siberian ibex. Similar low-overlap results were also recently confirmed in different Mongolian habitats (grasslands and alpine meadows [49];), including low overlaps between meso-carnivores and small mammals which are their prey.

In arid environments, most species face a trade-off between water requirements and conflicts associated with predation and competition [4, 22]. Waterholes are commonly associated with increased risks of predation [74]. However, for fulling the understanding of this conflict, it is necessary to begin to understand the behavioral and physiological flexibility for water requirements in the prey species [12]. This is especially evident in ungulates adapted to desert areas, where certain species, especially browsers, can extract water necessary for survival from their food [15, 37, 76]. This may be the reason for the low detection of goitered gazelle (only one record, despite the species being well-represented in the area [5];) which mostly lives in wide valleys far from mountain water sources. In the present study, only the argali used the waterholes all-year-round, while other large ungulates, like the khulan, showed clumped distributions peaking in the driest period (June–July) and a very low usage during the winter (see [56] regarding the influence of snow cover on the use of waterholes by the species). Wild Bactrian camel activity peaked in September (similar to the results previously reported by [77]) and Siberian ibex activity in November. Also, these desert-adapted ungulates may limit their activity during the day, to reduce heat loading and water loss. However, the Bactrian camel showed a uniform daily pattern, probably related to their ability to withstand thirst for extended periods [26] and the absence of natural predators (at least for adults [36];). Medium-sized herbivores, like the argali and Siberian ibex, only showed diurnal activity [77], although peaking at a different time, probably as an antipredator strategy, since both species showed seasonal, daily, and spatial segregation from their main predators. The khulan was the only large herbivore showing greater nocturnal activity (as also recently shown by [56]). In Xinjiang (China), a similar behavior of the species was also observed, although the pattern of waterhole use declined as the proximity to human settlements increased [78]. Finally, the tolai hare, the only non-ungulate herbivore, also without remarkable physiological mechanisms for decreasing water requirements, showed a nocturnal activity pattern all-year-round (to decrease water loss), and even a certain spatial overlap with a predator, the grey wolf, indicating that staying close to waterholes which support larger populations compared to other surrounding environments [16] is more important than avoiding predators. A similar overlap of the species with predators was recently observed by Mori et al. [49]. Altogether, the results show the great importance of waterholes for large and mid-size herbivores in the area all-year-round, since these are used in very different patterns by each species.

Regarding the predator species, high degrees of segregation have been observed in communities of carnivores in different environments [21, 55]. Similarly, only seasonal and daily overlaps were detected between the Eurasian lynx and snow leopard in the present study, while the grey wolf and red fox showed segregation at every studied level with the other carnivores in the area. The two felids (Eurasian lynx and snow leopard) were observed all-year-round, with nocturnal activity [44, 57]. Similarly, desert foxes are not physiologically well-adapted, and tend to behaviorally avoid heat stress, being active preferentially during the cooler hours of the night [27]. Finally, the grey wolf showed a seasonal activity peak around the middle of summer, and small dawn-dusk activity peaks, but still with moderate diurnal and nocturnal activity. They may become nocturnal to avoid human confrontation [70], and the current results in the absence of people confirms this behavioral flexibility of the species [1]. Pallas’s cat mainly distribute in steppe and semi-steppe habitats, and it was observed only once during the study, probably because the desert is not the most suitable habitat for them (however, see [7, 19] for other records within the area). Nonetheless, carnivores can also satisfy their water demand from food, reducing their dependency on water sources; thus, their activity patterns are mainly influenced by that of their prey.

The brown bear is the only omnivore in the area, and the only one hibernating, and thus its activity patterns deserve to be discussed independently. Furthermore, information about the activity patterns of the species is missing in current literature. The species emerged from hibernation in early March, showed a peak of activity around the first half of June which overlapped with the hottest period, and were less dependent on waterholes from mid-August until the start of hibernation. This autumn pattern may be related to their preference for *Nitraria* berries and *Ephedra* fruits during fall, which are good water sources [47]. Their activity was primarily nocturnal, probably avoiding the hot daytime conditions, since in many other environments the species is highly diurnal [52, 53].

In this predator-prey game around waterholes with low vegetation cover, the moon phase may have had a particular influence [58]. Only the snow leopard and Eurasian lynx (the species with a greater degree of activity overlap) showed an activity pattern moderately influenced by the lunar phase, suggesting a potential influence on predator-prey behavioral decisions [54]. However, the lowest influence of the moon phase was found among the prey species (herbivores). Certain correlations were also found between climatic conditions and activity patterns, but further data is necessary to disentangle the confounding effects of weather and season.

The results from the present study may be relevant for future management of the recorded species. A general pattern of seasonal, daily and/or seasonal segregation was observed in this undisturbed area but this peaceful equilibrium may be threatened in the future. In last few decades, an increasing rate of drought and severe winter conditions (locally known as ‘*dzud*’) have been observed in Mongolia [60]. The number of herders has increased in the buffer zone of the protected area over the past decades, and commonly when *dzud* occurred in these buffer zones, the Mongolian government granted livestock grazing rights in the area to local herders [24]. Human activities have a negative influence on the natural behavior of wildlife, due to direct competition for water and feed sources [2, 13, 41] and potential outbreaks of diseases, like rinderpest [8, 45]. Thus, once the natural (undisturbed) behavior of these species is described, as within this study, future efforts should focus on understanding the effects of livestock thereon. Moreover, climate change models predict increased temperatures, aridity and extended draught periods in the area [6, 18], which may provoke an increased overlap and may play a role in the increased transmission of infectious agents such as FPV, FCoV, CDV or rabies [63].

## Conclusions

These results provide a further understanding of the natural behavior of desert large and mid-sized mammal communities without human influence, and may help to improve integrated conservation strategies. Waterholes are key resource supporting the natural behavioral patterns of the community, and thus caution is recommended regarding the creation of additional water points without previously considering the specific needs of the species in the area and the potential effects on their predation-prey interactions.

## Data Availability

The datasets used and/or analyzed during the current study are available from the corresponding author on reasonable request.
